# Scoping Review: The Effectiveness of Interprofessional Virtual Reality Simulation

**DOI:** 10.1177/23821205251346327

**Published:** 2025-07-17

**Authors:** Nebras Alghanaim, Jo Hart, Gabrielle Finn

**Affiliations:** 1 Division of Medical Education, Faculty of Biology, Medicine and Health, 5292 University of Manchester, Manchester, UK; 2 Faculty of Medicine, 48149 King Saud bin Abdulaziz University for Health Sciences, Jeddah, Saudi Arabia; 3 King Abdullah International Medical Research Center, Jeddah, Saudi Arabia; 4 Ministry of the National Guard- Health Affairs, Jeddah, Saudi Arabia

**Keywords:** interprofessional education, virtual reality, simulation

## Abstract

**Background:**

Interprofessional education (IPE) is utilised as a teaching strategy for future health practitioners to be effective team members in the healthcare system. Technology such as simulation is widely used in education. Virtual reality (VR) is a branch of simulation where learners interact in a virtual world. This scoping review aimed to evaluate the effectiveness of virtual reality simulation modality in interprofessional (VR-Sim IPE) health education schemes, specifically for non-technical skills.

**Method:**

The PRISMA-ScR checklist and the Arksey and O’Malley methodological framework were used. Databases searched were Web of Science, ProQuest, OVID, SCOPUS, CINAHL Plus and British Educational Index from 2010 to 2025. Twelve articles met the search criteria. Articles were reviewed using three high-quality assessment tools: MERSQI, CASP and MMAT.

**Results:**

To explore effectiveness, four models were used. Firstly, findings were mapped to the four IPE domains/capabilities: Ethical practice, knowledge in practice, interprofessional working and reflection (learning). The second model was the 6 levels of learning efficacy; most studies showed level 4 of learning efficacy, while few articles captured the highest level (level 6). Thirdly, according to Revised Bloom's taxonomy learning domain (specifically the knowledge-cognitive dimension), 42% of the articles were categorised in analysing cognitive level and conceptual knowledge dimension. The highest level detected was evaluating cognitive level and procedural knowledge dimension. Finally, the modified Kirkpatrick's model for evaluating the effectiveness of educational outcomes was detected in 42% of the studies at level 3, ie, change in behaviours, the highest level captured in the review.

**Discussion and conclusion:**

This study showed how VR might be used in IPE activities. None of the articles assessed a procedure's technical skills or algorithm. Future considerations of implementing IPE using VR in undergraduate health students’ modules and utilising the VR in assessment, for example, formative or summative objective structured clinical examinations (OSCEs), are required.

## Introduction

Interprofessional education (IPE) has been defined by the Centre for the Advancement of Interprofessional Education (CAIPE) as ‘when two or more professions learn with, from and about each other to improve collaboration and quality of care’.^1^ The concept of IPE in the health field started in the 1970s in the UK, and it has been emphasised as one of the teaching requirements in the Health and Personal Social Services Programmes report by promoting multi-disciplinary team training.^2^ Since then, the World Health Organisation (WHO) has been crucial in extending IPE globally.^3^ IPE played a significant role in enhancing patient safety and minimising medical errors^4,5^ and was addressed in the latest Institute of Medicine (IOM) report^6^ on IPE published in 2015. It underscored the importance of professionals from various disciplines collaborating to improve patient outcomes. The report also proposed methods to assess how IPE affects collaborative practice and health system results. It emphasised the necessity for well-conceived studies to establish a connection between IPE and improvements in patient care, safety and satisfaction. The IOM's emphasis on IPE has spurred the development of interprofessional programmes in health education worldwide.

Healthcare simulation is widely acknowledged as an excellent tool for IPE. It plays a role in increasing awareness of the importance of IPE in clinical care, being active team members and having perspective towards interprofessional work^7–10^ in a safe, controlled environment. Simulation-enhanced interprofessional education (Sim-IPE) is one of the INACSL standards of best practice, which allows learners from different professions to be involved in a simulation experience to achieve shared or linked goals and outcomes.^11^ Simulation is the creation of a realistic environment of a complex real world. The goal of simulation is to promote learning through engagement, reflection, feedback and practice without the risk associated with a similar real-life experience.^12^ Simulation plays a crucial role in revamping the medical education curriculum that promotes experiential learning, with an emphasis on the cognitive, psychomotor and affective learning domains of Bloom's taxonomy.^12^

Virtual reality (VR), in the last decade, has progressed from science technology to being a potentially beneficial tool for health educators.^13^ VR is defined as a ‘computer or internet-based learning environment that includes virtual worlds with the use of avatars’.^,^^14^^, p.6^69 VR has been widely utilised in nursing and medical disciplines, allowing students to learn via virtual experience.^13^ In VR sessions, learners don a virtual reality headset and immerse themselves in an interactive virtual environment. In contrast to other simulation typologies, VR occurs asynchronously whenever and wherever the learner prefers. The virtual world setting can be changed to fit instructional objectives, allowing incorporation into learning. Although initial content production and equipment costs may be costly, VR's scalability and flexibility can result in long-term cost savings and an excellent cost-utility ratio compared to traditional simulation modalities.^15^ Three examples have demonstrated the cost-effectiveness of VR in the field of health education, underscoring its potential to enhance learning outcomes and provide scalable solutions.^16–18^ A systematic review and meta-analysis of healthcare education revealed that VR significantly improves knowledge and skill acquisition compared to traditional methods, establishing it as a cost-effective and practical alternative for delivering curriculum, especially in resource-limited settings.^16^ Additionally, a comprehensive scoping assessment explored the integration of VR into medical education, highlighting that, notwithstanding initial expenditures, virtual reality affords authentic learning experiences and exhibits scalability as an alternative to conventional instruction.^17^ A pilot study on low-cost VR tools for medical education found that VR effectively increases patient exposure and enriches the curriculum, thereby solidifying its potential as a cost-effective training resource.^18^

VR is becoming increasingly popular as a teaching methodology in health professional education.^19^ It has been successfully used in continuing medical education for practising physicians, nurses and other allied health professionals, as it is an excellent tool for teaching clinical skills and enhancing clinical decision-making.^15^ VR provides an immersive and engaging learning environment for simulated clinical situations.^20^ VR researchers believe that using avatars and virtual simulators to train healthcare providers is a viable option. VR was created to assist students and professionals in polishing their workplace skills through purposeful practice.^19^ VR does have some disadvantages in education, including the high initial cost of VR, the haptic issue and reduced face-to-face real communication, which may be one of the main limitations.^21^ Also, incorporating VR in IPE encounters particular challenges that need to be addressed to ensure its efficacy. A study on VR simulation-based IPE identified multiple problems,^22^ specifically, the unfamiliarity with virtual worlds among educators and students may result in discomfort and decreased engagement, alongside inconsistencies in student participation during interprofessional education sessions. Additionally, there are difficulties in facilitating within interprofessional groups. Successfully managing group dynamics in a VR environment necessitates specific skills and facilitators commonly encounter challenges in overseeing virtual interactions. Moreover, implementation expenses continue to pose a significant challenge.

In healthcare education, numerous frameworks and theories evaluate teaching and learning modalities and interventions. It is important to understand the differences between assessing competencies and capabilities. The Centre for International Development and Training defines competency as ‘the actual levels of skills, knowledge and attitudes’.^23^ It signifies the state of being qualified to perform a task adequately, ie, at the individual level. Competency denotes specific, measurable skills and behaviours necessary for effective task performance.^24^ In contrast, capability is described as ‘higher than competency because it pertains to the deployment and application of competencies’, ie, at the organisational or institutional levels.^23^ In other words, capability encompasses a more extensive integration of skills involving the adaptation to new situations and the application of those competencies in unfamiliar, evolving environments and unforeseen circumstances.^24^ Capability represents the ability and capacity to achieve and sustain a desired outcome.^24–26^ This distinction highlights the need to cultivate capable learners who can navigate complex and shifting environments, such as IPE, rather than merely concentrating on discrete competencies.^24–26^

This difference is vital in IPE, as it emphasises that health professionals must possess not only specific competencies but also the ability to collaborate across diverse settings and challenges, such as simulation. There are four well-known IPE frameworks^24^: the Interprofessional Capability Framework,^27^ Core Competencies for Interprofessional Collaborative Practice,^28–30^ the National Interprofessional Competency Framework^31^ and the Curtin University Interprofessional Capability Framework.^32^ From a comparative review analysis,^24^ while all four frameworks aim to enhance collaborative practice among health professionals, the Interprofessional Capability Framework provides a holistic and inclusive approach to fostering IPE.^24^ It aligns well with global healthcare trends, making it a superior choice for building resilient, capable and ethical health professionals.^24^ Furthermore, it places a strong emphasis on fostering teamwork and partnerships rather than merely prescribing specific roles or actions.^24^ This adaptability renders it particularly suitable for worldwide healthcare systems, where flexibility and shared decision-making are essential. Virtual reality simulation in interprofessional education (VR-SIM IPE) serves as an ideal method to implement and evaluate these capabilities in a controlled, experiential learning environment. By integrating the Interprofessional Capability Framework into simulation, healthcare programmes can offer learners hands-on opportunities to develop the core capabilities needed for effective interprofessional practice.

Health education often requires learners to progress through these stages to ensure they not only understand health concepts but can also apply them effectively in diverse and unpredictable situations. A prominent model that describes the cognitive processes of learning and development in the context of health education is Revised Bloom's Taxonomy.^33,34^ It offers a comprehensive framework for designing and evaluating educational programmes. It categorises learning objectives into two main dimensions: the knowledge dimension and the cognitive process dimension, which guide learners through a hierarchy of cognitive processes, from foundational knowledge to complex problem-solving.^33,34^ This framework provides a structured approach to understanding how learners acquire, process and apply knowledge, making it highly relevant to health education. For example, within the VR-SIM IPE context, students begin by remembering and understanding the roles and responsibilities for effective interprofessional teamwork. While in simulation, they apply interprofessional communication strategies and clinical skills in controlled environments.

Furthermore, it is essential to evaluate and differentiate between efficacy and effectiveness in innovative approaches,^35^ such as VR-SIM IPE. In health education, efficacy refers to the ability of an intervention or educational programme to achieve desired positive effects in ideal or controlled settings. It assesses whether the intervention can attain its intended goals when implemented as planned. Understanding efficacy is crucial in health education, as it helps evaluate the potential impact of educational strategies before their wider implementation.^35^ One of the widely recognised frameworks for assessing efficacy is the learning efficacy model.^36^ This model evaluates how effectively educational strategies support desired learning outcomes, which is vital in preparing healthcare professionals for collaborative practice.^36^ By utilising the learning efficacy model within VR-SIM IPE contexts, educators can assess and enhance their teaching strategies, ensuring they effectively develop the interventions needed for collaborative healthcare practice.

It is important to distinguish efficacy from effectiveness, which measures how well the intervention functions in real-world settings.^35^ Effectiveness denotes how an intervention achieves its intended health outcomes within everyday practice environments.^35^ For health educators and stakeholders, understanding effectiveness is crucial, as it reflects the performance of a health education programme in standard community or clinical contexts.^35^ To evaluate effectiveness, it is essential to consider several factors, including the programme's adaptability to various populations, the practicality of its implementation and its long-term sustainability.^35,37^ The modified Kirkpatrick's model is one of the most significant and widely used frameworks for assessing effectiveness in health education.^37,38^ By applying Kirkpatrick's model in the context of VR-SIM IPE, educators can evaluate the effectiveness alongside adaptations, ensuring that these interventions not only enhance learning but also lead to improved collaborative behaviours and patient care outcomes. These models emphasise a dynamic approach, focusing on the critical role of assessing complex and evolving healthcare contexts such as VR-SIM IPE.

The aim of this scoping review was to appraise and synthesise the best evidence on the effectiveness of virtual reality simulation in interprofessional education (VR-Sim IPE**)** for health professions, specifically non-technical skills. The data from included studies were synthesised to evaluate the efficacy of interprofessional virtual reality simulation. The objectives of this work are as follows:

1. To explore the influence of interprofessional virtual reality simulation on interprofessional capabilities.
2. To identify the impact of interprofessional virtual reality simulation on learners’ cognitive process dimension.
3. To recognise the interprofessional virtual simulation learning efficacy level as a teaching modality.
4. To measure the effectiveness of interprofessional virtual reality simulation on learning outcomes.

## Method

### Conceptual Framework

#### Research paradigm

In this review, a postpositivist paradigm that integrated both quantitative and qualitative approaches to measure the effectiveness of interprofessional virtual reality simulation^39^ as a mix of quantitative and qualitative educational outcomes enhanced the review findings. Mixed methods were used to improve accuracy. The value of the mixed method is to get a wider picture of phenomena without biases.^40^ The post-positivist philosophical viewpoint is a dynamic research approach that enables the investigator to employ several techniques for searching. It allows for the utilisation of many research methodologies to ensure that the issue is explored from multiple angles, and therefore, tends to minimise personal preconceptions.^39,41^ Post-positivism promotes the triangulation of qualitative and quantitative data from a diverse range of facts and respects all values to develop knowledge.^42^ Thus, in this postpositivist educational review, pre-post studies, rating scales, measurements, observations, interviews, surveys and exam performance were included in the search extraction,^42^ in line with its theoretical lens.

#### Theoretical underpinning of interprofessional virtual reality simulation

This review includes two concepts of educational strategies: interprofessional education and VR Simulation. It aligns with transformative learning theory. Transformative learning theory originates from the premise that adults need to enhance their abilities to become an ‘independent autonomous thinker’.^43^ The major foci of this theory occur when learners develop their appreciation of the world through reflection on experiences.^44^

It is promoted by such methods as group projects, role-play, case studies and simulation.^43^ The key is to enable learners to become involved with the concept presented in the context and collectively critically assess the justification of new knowledge.

Virtual reality simulation in health education plays an important role in transformative learning as learners develop their self-reflection, assessment of clinical reasoning, performance assessment, beliefs, attitudes and cognitive schema that enable them to work in the complex health context, as using VR enables them to consider more critically on how they would apply their newfound knowledge to their professional career.^45^ However, transformative learning, on the other hand, enables IPE as well for a better understanding of the need for a new way of thinking. This is based on the IPE requirement of appreciation of different approaches to learning^44^ through interactions with others, environment, reflection on these experiences and thus working collaboratively to create new learning.

### Methodological Framework

Scoping reviews builds the knowledge of existing literature and gives a sufficient view of the topic.^46^ There has been an increase of scoping reviews in the field of health profession education in the past two decades^47–49^ by 4200%,^50^ as they map the depth and breadth of developing areas in medical education, grasp how research is conducted on a subject and reveal the various types of evidence that exist regarding it.^47–49^ Scoping reviews enhance this by aggregating evidence, clarifying concepts and identifying emerging trends in the literature by employing predefined criteria and frameworks to map and categorise literature, thus providing a comprehensive and unbiased overview of research findings across various study designs and methodologies.^51^

To develop a rigorous scoping review and to ensure reliability, the preferred reporting items for systematic review and meta-analyses extension for scoping review (PRISMA-ScR) checklist^52^ was used in developing and reporting the study (checklist added as Supplemental material for this article), as well as the Arksey and O’Malley six steps methodological framework: (1) identifying the research question, (2) searching for relevant studies, (3) selecting studies, (4) charting the data, (5) collecting, summarising and reporting the results, and (6) consulting with stakeholder optional.^51,53^ In this review, step 6 was not used.

(1) Identifying the research question

The main question is: To what extent is interprofessional virtual reality simulation an effective teaching modality, and how does it affect learner's non-technical skills?

Also, this scoping review was designed to address the following subsidiary questions:

1. How does interprofessional virtual reality simulation influence students’ interprofessional capabilities?
2. How effective is interprofessional virtual reality simulation in health professions?
3. How does interprofessional virtual reality simulation impact learners’ knowledge and cognitive complexity levels according to the (a) Revised Bloom's Taxonomy knowledge and (b) Cognitive process dimensions?
4. What is the effectiveness of interprofessional virtual reality simulation on learning outcomes?

(2) Searching for relevant studies

Reviewer:

Extraction was conducted by the first author as part of their doctoral studies. However, a sample was reviewed and discussed by all authors. Extraction and discussions were iterative in nature.

Protocol and registration:

The reviewer searched the International Prospective Register of Systematic Reviews (PROSPERO)^54^ and no existing systematic reviews were found that fit the inclusion criteria. This study protocol is not registered in PROSPERO, as in order to be registered, studies need to contain at least one outcome of direct patient or clinical relevance.

Search strategy:

Search terms and subject headings were: ‘(Undergrad* OR Postgrad*) AND (Interprofession*OR interprofessional learning OR interprofessional collaboration OR Health profession*) AND (Simulation based OR interprofessional simulation OR interprofessional virtual reality simulation) AND (Virtual reality OR Virtual simulation) AND (Team based competenc*OR Interprofessional competenc* OR Skill*)’. The review covered English language empirical studies from 2010 to 2022. The review included six databases that are considered the largest and most commonly used in health education research, including Web of Science, ProQuest, OVID, SCOPUS, CINAHL Plus and British Educational Index BEI. The updated search from 2022 to February 2025 covered the same databases and search terms.

Data extracted included date, author(s), study design, country, participants, methods (intervention and tools) and quantitative/ qualitative outcomes (see Table 2). Definitions for IPE, VR and SIM were delineated in the introduction of this study.

**Table 1.** Summary of educational models.

Educational models	
Interprofessional capabilities^27^	Knowledge in practice: describes how non-judgmental practice informs a patient-centred participatory service by capturing awareness of others’ professional regulations in the interprofessional team. Ethical practice: focuses on encouraging patient participation in the interprofessional team's decision-making processes and the need for practitioners to be sensitive to the demands made in the law by other professions regarding their duty of care and the underlying ethos of the various professional groups. Interprofessional working: encompasses patient-centred engagement, assessment and communication tactics, as well as the ability to identify and work toward mutual adaptation between the patient/user and the team. Reflection (learning): identifies the development of reciprocity across professions, the use of evidence-based practice, and the integration of continuous professional development and supports this crucial part of contemporary practice.
Revised Bloom's Taxonomy^34^	Factual knowledge: students must understand fundamental aspects to become familiar with a discipline or solve its difficulties. Conceptual knowledge: the interconnections between the essential pieces of a more extensive system allow learners to work together. Procedural knowledge: how to do something; investigation methods and criteria for employing skills, algorithms, procedures and methodologies. Metacognitive knowledge: awareness and knowledge of one's own cognition and knowledge of cognition in general.
Learning efficacy^36^	Low strength of evidence: mostly practitioner wisdom or expert opinion, anecdotal or poorly constrained qualitative or quantitative data supporting the application of the strategy to improve student learning. Low strength of evidence: mostly practitioner wisdom or expert opinion; may have some case or cohort studies, mixed results with learning gains in some settings but not in others or improved learning with infrequent application of strategy under review. Moderate strength of evidence: at least one case or cohort study conducted on the strategy, evidence of learning gains in one setting (classroom, field, or lab) or inconsistent evidence of learning gains in two or more settings or disciplines. Moderate strength of evidence: at least one case or cohort study conducted on the strategy, consistent evidence of learning gains in two or more settings (classroom, field or lab) or disciplines. High strength of evidence: multiple cohorts or higher-level studies conducted on the strategy, evidence of consistent learning gains in one type of setting (classroom, field or lab) or inconsistent evidence of learning gains across two or more settings or disciplines. High strength of evidence (multiple cohorts or higher-level studies conducted on the strategy), consistent evidence of learning gain in two or more settings (classroom, field, or lab) or disciplines.
Modified Kirkpatrick's model ^38^	Level 1 - Reaction: Participants’ views on the learning experience, its organisation, presentation, content, teaching methods and quality of instruction. Level 2A - Learning, change in attitudes: Changes in the attitudes or perceptions among participant groups towards teaching and learning. Level 2B - Learning, modification of knowledge or skills: For knowledge, this relates to the acquisition of concepts, procedures, and principles; for skills, this relates to the acquisition of thinking/problem-solving, psychomotor, and social skills. Level 3 - Behaviour, change in behaviours: Documents the transfer of learning to the workplace or willingness of learners to apply new knowledge and skills. Level 4A - Results, change in the system/organisational practice: Refers to wider organisational changes attributable to the educational programme. Level 4B - Results, change among the participants, students, residents or colleagues: Refers to improvement in student or resident learning/performance as a direct result of the educational intervention.

**Table 2.** Descriptive summary of included studies from 2010 to 2025.

Study	Demographics (i) Study design (ii)Participants (iii) Country	Methods (i) Intervention (ii) Tool	Results (i) Quantitative outcomes (ii) Qualitative outcomes	Educational models
(Neher et al, 2024)^66^	**(i) Study design** Pre-post study **(ii) Participants** Final year medical (n = 21) and nursing student (n = 21) **(iii) Country** Switzerland	**(i) Intervention** Interprofessional team (INTEAM) training course that included a VR simulation (Sim X) of a neurological emergency case (seizure). Scheduled for 3 consecutive days, with each day consisting of 3 separate 3-h time slots. 6 students were invited per slot (3 nursing and 3 medical students), as had 3 rooms and 3 moderators’ available. **(ii) Tool** - Handover Assessment Tool using ISBAR - Self-constructed questionnaire for confidence. - Training Evaluation Inventory	**(i) Quantitative Outcomes:** **Handover Assessment Tool using ISBAR:** Participants’ ability to perform structured patient handovers showed a significant enhancement post-training. Median scores increased from 8 (IQR 6-9) before training to 8 (IQR 7-9) after training, indicating a moderate effect size (*r* = .33; *P* = .045). **Self-constructed questionnaire:** Students’ confidence in caring for patients experiencing seizures increased significantly. For caring for patient with seizure subscale: Median confidence levels rose from 3 (IQR 2-3) pre-training to 3.5 (IQR 3-4) post-training, reflecting a large effect size (*r* = .60; *z* = −3.8; *P* < .001) students felt more capable of evaluating and decisions-making. For Recognising when to call for help subscale median confidence levels increased significantly from 4 (IQR 3-4) pre-training to 4 (IQR 4-4) post-training, reflecting a moderate to large effect size (*r* = 0.47; *P* = .003). No statistically significant differences between pre and post-test for the ‘working interprofessionally’ median 4 (IQR 4-4) and 4 (IQR4-4) (*P* = .87). But Medical students indicated lower confidence than nursing students at pretest for the item ‘working interprofessionally’ (z = −2.3; *P* = .02; *r* = .36). **Training Evaluation Inventory:** The results of the Training Evaluation Inventory indicate a high perceived effectiveness. The sub-scores subjective enjoyment, perceived usefulness and attitudes toward training were rated high (median 5, IQR 4-5). The sub-scores perceived difficulty and subjective knowledge gain received medium ratings (median 4, IQR 4-5 and median 4, IQR 3-5, respectively).	**IPE domain/capabilities:** Knowledge in practice Ethical practice **Knowledge/cognitive Process Dimension:** Evaluate and conceptual knowledge **Learning efficacy:** Level 6: High strength of evidence. **Modified Kirkpatrick's Model:** **Level 3:** Behaviour - Change in behaviours
(Tschannen et al, 2018)^68^	**(i) Study design** Pre-post design **(ii) Participants** Total of (*n* = 41) Nursing (*n* = 6) Medicine (*n* = 15) Pharmacy (n = 18) Social worker (*n* = 2) **(iii) Country** USA	**(i) Intervention** Applying 3 scenarios using ‘Second life’ Virtual Reality platform. **(ii) Tool** 10 item surveys	**(i) Quantitative outcomes:** Post-knowledge test scores improved as evidenced by a mean score of 9.63 (SD = 0.58) for all disciplines. Developed new skills and/or knowledge and intended to use this gained knowledge and skills in clinical area: Mean ± SD (4.9 ± 0.32).	**IPE domain/capabilities:** Ethical in practice, knowledge in practice, interprofessional working. **Knowledge/cognitive process dimension:** Analyse and conceptual knowledge **Learning efficacy:** Level 4: Moderate strength of evidence **Modified Kirkpatrick's Model:** **Level 2B:** Learning - Modification of knowledge or skills.
(Zook et al, 2018)^67^	**(i) Study design** Pre-post design **(ii) Participants** Total of (*n* = 26) Nursing (*n* = 8) Psychology (*n* = 9) Speech language pathology (*n* = 9) **(iii) Country** USA	**(i) Intervention** Multi (3) semester scaffolded interprofessional curriculum, 4 virtual simulation scenarios. **(ii) Tool** Interprofessional Socialization and Valuing Scale (ISVS).	**(i) Quantitative outcomes:** Over three terms: Statistically significant effect on ‘self-perceived ability to work with others’ sub-scale (*F* = 8.37, *P* < .001), and ‘comfort in working with others’ sub-scale (*F* = 3.96, *P* < .01). Not statistically significant on ‘value of working with other’ sub-scale (*F* = 2.16, *P* = .114).	**IPE domain/capabilities:** Interprofessional working. **Knowledge/cognitive process dimension:** Apply and conceptual knowledge. **Learning efficacy:** Level 4: Moderate strength of evidence **Modified Kirkpatrick's Model:** **Level 2A:** Learning - Change in attitudes.
(Sweigart et al, 2016)^69^	**(i) Study design** Pre-post design **(ii) Participants** Total of (*n* = 92) Nursing (*n* = 45) Medicine (*n* = 13) Occupational therapy (*n* = 27) Social work (*n* = 7) **(iii) Country** USA	**(i) Intervention** Virtual platform. **(ii) Tool** The TeamsSTEPPS Teamwork Attitudes Questionnaire (T-TAQ).	**(i) Quantitative outcomes** Significant positive changes in four sub-scales: 1. ‘Leadership’ sub-scale. 2. ‘Situation monitoring’ sub-scale. 3. ‘Mutual support’ sub-scale. 4. ‘Communication’ sub-scale.*P* values (from .001 to .002).‘Team structure’ was statistically not significant.	**IPE domain/capabilities:** Ethical in practice, Interprofessional working. **Knowledge/cognitive process dimension:** Apply and conceptual knowledge. **Learning efficacy:** Level 4: Moderate strength of evidence. **Modified Kirkpatrick's Model:** **Level 2A:** Learning - Change in attitudes.
(Liaw et al, 2020)^73^	**(i) Study design** An exploratory, descriptive qualitative study **(ii) Participants** Total of (*n* = 16) Nursing (*n* = 5) Medicine (*n* = 7) Physiotherapy (*n* = 2) Social worker (*n* = 2) **(iii) Country** Singapore	**(i) Intervention** IPE virtual reality simulation (Mental model). **(ii) Tool** Three focus groups	**(ii) Qualitative outcomes:** Three themes: 1. Gaining insight into mutual roles. 2. Seeing the patient as a whole. 3. Gap in real-world application.	**IPE domain/capabilities:** Ethical practice, knowledge in practice, reflection (learning) **Knowledge/cognitive process dimension:** Analyse and conceptual knowledge **Learning efficacy:** Level 4: Moderate strength of evidence **Modified Kirkpatrick's Model:** **Level 2B:** Learning - Modification of knowledge or skills
(Tran et al, 2020)^71^	**(i) Study design** Qualitative **(ii) Participants** Total of (*n* = 39) Nursing (*n* = 16) Medical (*n* = 12) Physiotherapy (*n* = 4) Occupational therapy (*n* = 7) **(iii) Country** Sweden	**(i) Intervention** 10 VR sessions in Primary healthcare module. Survey after the session and group interview/ **(ii) Tool** Qualitative content analysis and inductive approach.	**(ii) Qualitative outcomes:** Four themes: 1. VR facilitates the learning process. 2. Benefits of multi-disciplinary working. 3. Understand roles and competencies of other professions. 4. All needed in clinical work	**IPE domain/capabilities:** Ethical Practice, knowledge in practice, interprofessional working, reflection (learning) **Knowledge/cognitive process dimension:** Analyse and conceptual knowledge **Learning efficacy:** Level 4: Moderate strength of evidence **Modified Kirkpatrick's Model:** **Level 2B:** Learning - Modification of knowledge or skills
(Williams et al, 2020)^70^	**(i) Study design** Descriptive qualitative study pre-post questionnaire. **(ii) Participants** Total of (*n* = 46) Bachelor of Science in nursing (*n* = 27) Practical nursing (n = 12) Health care assistant (n = 7) **(iii) Country** Canada	**(i) Intervention** Using the ‘Clinispace’ VR platform. Semi-structured questionnaire based on CIHC framework. **(ii) Tool** Directed content analysis	**(ii) Qualitative outcomes:** Three themes: 1. Intentional collaboration 2. Role awareness 3. Position of power	**IPE domain/capabilities:** Ethical practice, knowledge in practice, interprofessional working. **Knowledge/cognitive process dimension:** Apply and conceptual knowledge **Learning efficacy:** Level 4: Moderate strength of evidence. **Modified Kirkpatrick's Model:** **Level 2A:** Learning - Change in attitudes.
(Shoemaker et al, 2014)^72^	**(i) Study design** Retrospective qualitative case report **(ii) Participants** Total of (*n* = 100) Physician assistant (*n* = 30) Physiotherapy (*n* = 46) Occupational therapy (*n* = 24) **(iii) Country** USA	**(i) Intervention** Virtual patient **(ii) Tool** Collaborative thematic analysis	**(ii) Qualitative outcomes:** Three themes: 1. Benefits of collaborative care. 2. Role clarification 3. Relevance for future practice.	**IPE domain/capabilities:** Ethical practice, knowledge in practice. **Knowledge/cognitive process dimension:** Evaluate and procedural knowledge. **Learning efficacy:** Level 6: High strength of evidence **Modified Kirkpatrick's Model:** **Level 3:** Behaviours
(Lee et al, 2019)^76^	**(i) Study design** Pre-post design & Student reflective **(ii) Participants** Total of (*n* = 35) From two institutions Medicine (*n* = 12) Nursing (*n* = 6) Nutrition (*n* = 7) Physiotherapy (*n* = 5) Social work (*n* = 5) **(iii) Country** USA	**(i) Intervention** 10 VR palliative care sessions. **(ii) Tool** Quantitative: Five validated scales 1. The Attitudes Towards Health Care Team Survey (ATHCT) 2. The Readiness for Interprofessional Learning Scale (RIPLS) 3. The Interprofessional Collaboration Scale (ICS) 4. The Team Skills Scale (TSS) 5. The team Fitness Test (TFT) Qualitative: 1. Reflective writing reviewed and coded through an open coding immersion/crystallisation.Photovoice analysis for photographs using a phenomenological inquiry approach.	**(i) Quantitative Outcomes** Statistically significant improvement in post-test: 1. AHCT:‘Team value’ sub-scale *P* = .019 and ‘team efficiency’ sub-scale *P* = .048 2. RIPLS:‘Teamwork and collaboration’ sub-scale *p* = 0.034 and ‘positive professional identity’ sub-scale *p* = 0.019. 3. ICS:‘Accommodation’ sub-scale *p* = 0.005 and ‘isolation’ sub-scale *p* = 0.035. 4. TSS:‘Leadership communication skills’ sub-scale *p* = 0.019 and ‘technical/ professional abilities’ sub-scale *p* = 0.009. 5. TFT:not statistically significant for all. **(ii) Qualitative outcomes:** Four themes: 1. Value of IPE team 2. Comfortable learning environment 3. Learning unique and engaging learning experience. 4. Technology mediated learning experience	**IPE domain/capabilities:** Ethical practice, knowledge in practice, reflection (learning). **Knowledge/cognitive process dimension:** Analyse and conceptual knowledge. **Learning efficacy:** Level 6: High strength of evidence. **Modified Kirkpatrick's Model:** **Level 3:** Behaviours
(Liaw et al, 2019)^75^	**(i) Study design** Pre-post design & Focus group. **(ii) Participants** Total of (*n* = 29) From three institutions Medicine (*n* = 6) Nursing (*n* = 6) Pharmacy (*n* = 4) Physiotherapy (*n* = 6) Occupational therapy (*n* = 6) Social worker (*n* = 1) **(iii) Country** Singapore	**(i) Intervention** Two scenarios over three days using the ‘3D-VE’ platform. **(ii) Tool** Quantitative: Five validated scales: 1. Attitudes toward Interprofessional Health Care Teams (ATHCT) 2. Interprofessional Socialisation and Valuing Scale (ISVS) 3. System Usability Scale (SUS) 4. Sociability of Computer-Supported Collaborative learning scale 5. French-Canadian adaptation questionnaire Qualitative: Four focus groups.	**(i) Quantitative outcomes** 1. ATHCT is statistically higher in post-test overall(*P* < .05) 2. ISVS improvement in interprofessional competencies and attitude toward interprofessional team (*P* < .001) 3. SUS: Overall positive usability of 3D-VE (mean 3.48, SD 0.64). 4. Sociability of Computer-Supported Collaborative learning scale overall score (mean 3.39, SD 0.60) positively about 3D-VE. 5. French-Canadian adaptation questionnaire shows a moderate sense of presence in 3D-VE with 107 out of 168 (SD17.78). **(ii) Qualitative outcomes:** Four themes: 1. Feeling real 2. Less threatened, feeling able to hide, less stress and react confidently. 3. Understanding each other's roles and acknowledging different professional priorities in patient care. 4. Technical hiccups	**IPE domain/capabilities:** Ethical practice, reflection (learning) **Knowledge/cognitive process dimension:** Evaluate and conceptual knowledge. **Learning efficacy:** Level 6: High strength of evidence. **Modified Kirkpatrick's Model:** **Level 3:** Behaviours
(Martini et al, 2019)^74^	**(i) Study design** Pre- post design & observation and interview **(ii) Participants** Total of (*n* = 40) Medical (*n* = 20) Pharmacy (*n* = 20) **(iii) Country** New Zealand	**(i) Intervention** 10 Paired VR session (*n* = 20) Medicine *n* = 10 Pharmacy *n* = 10 10 Non-paired VR Session (*n* = 20) Medicine *n* = 10 Pharmacy *n* = 10 **(ii) Tool** Quantitative: The Readiness for Interprofessional Learning Scale (RIPLS). Qualitative: Semi-structured interview and structured observation.	**(i) Quantitative outcomes** From paired sim: Statistically improvement in the ‘teamwork and collaboration’ domain (*P* = .006) and ‘positive professional identity’ domain (*P* = .0003) And overall improvement in attitudes toward interprofessional learning. **(ii) Qualitative outcomes:** Observation themes: Professions tend to start communicating with the patient. Students were discussing before making an action plan. Interview themes: 1. Confidence in clinical decision-making 2. Teamwork-shared decision making. 3. Communicating thought processes 4. Appreciation of roles and responsibilities 5. Attitudes to the simulation and IPL.	**IPE domain/ capabilities:** Ethical Practice, interprofessional working, reflection (learning). **Knowledge/cognitive process dimension:** Analyse and conceptual knowledge. **Learning efficacy:** Level 4: Moderate strength of evidence **Modified Kirkpatrick's Model:** **Level 2B:** Learning - Modification of knowledge or skills
(Caylor et al, 2015)^77^	**(i) Study design** Pre-post design & observation **(ii) Participants** Total of (*n* = 21) Nursing (*n* = 8) Pharmacy (*n* = 7) Medical (*n* = 6) **(iii) Country** USA	**(i) Intervention** VR session using second life. **(ii) Tool** Quantitative: 4 validated instruments: 1. The Interdisciplinary Education Perception Scale (IEPS) 2. Team STEPPS Teamwork Attitudes Questionnaire (T-TAQ) 3. Teamwork STEPPS Team Performance Observation Tool(T-TPOT) 4. Technology and Overall Experience Survey (TOES)Qualitative:Observation.	**(i) Quantitative outcomes:** Not reporting specific significant results. Overall improvement in T-TAQ and IEPS. TOES suggested second life as an effective platform. **(ii) Qualitative outcomes:** Observation themes: 1. Ability to identify each other roles. 2. Discussing the importance of action accountability. 3. Demonstrate profession-specific input.	**IPE domain/capabilities:** Ethical practice, knowledge in practice, interprofessional working. **Knowledge/cognitive process dimension:** Apply and conceptual knowledge. **Learning efficacy:** Level 4: Moderate strength of evidence **Modified Kirkpatrick's Model:** **Level 2A:** Learning - Change in attitudes

(3) Selecting studies

Criteria for included studies:

The PEO (participants, education, outcome) model is an acceptable model of interprofessional education and is widely used.^55^ Accordingly, this scoping review adapted the PEO model.

- Participants: Any two or more healthcare students at undergraduate or postgraduate level.
- Education: Any virtual reality simulation scenarios/sessions.
- Outcome: Any objectively or subjectively non-technical skills measured educational outcome related to interprofessional virtual reality simulation assessed by quantitative assessment through validated instruments or qualitative data, if any, were considered.

Criteria for excluded studies:

Uni-professional education, any non-health-related professions, hybrid simulation or augmented reality studies were excluded as they were out of the review scope. Technical skills and procedural algorithms were also excluded. Non-English language journals and grey literature, ie, unpublished work or work published in non-commercial forms, including reports and working papers, were also excluded.

Critical appraisal of individual sources of evidence:

Assessment of methodological quality (quantitative):

The Medical Education Research Study Quality Instrument (MERSQI) was used to evaluate quantitative studies retrieved in the review. This reliable and valid instrument tool was created to assess the methodological quality of medical education research and debate its utility in evaluating educational studies. This has been used to evaluate the quality of various quantitative educational studies.^56,57^ It has ten components representing six domains: study design, sample, data type, validity, analysis and outcomes. Each domain may include more than one component to be scored, and each component marks independently (0, 0.5, 1, 1.5, 2, 3); the highest score in each domain is 3, and thus, the highest study score is 18.

Assessment of methodological quality (qualitative):

The Critical Appraisal Skills Programme (CASP) qualitative study review checklist^58^ assessed qualitative studies’ adequacy, quality and rigour. CASP has been endorsed by the Cochrane Qualitative and Implementation Methods Group to assess if the study methods are appropriate and whether the findings are well-presented and meaningful.^59^ It is the most widely used checklist/criteria-based instrument for quality appraisal in health and social care-related qualitative evidence synthesis and is recommended for novice researchers.^59^ It covers three main questions: Are the results of the study valid? What are the results? Will the results help locally? Recorded answers would be ‘yes’, ‘no’ or ‘can't tell’.^58^

Assessment of methodological quality (mixed methods):

Mixed methods appraisal tool (MMAT) was created to evaluate systematic mixed studies. The MMAT can be employed to assess empirical research, ie, primary data. Non-empirical papers, such as reviews and theoretical papers, cannot be used with it. It divides methodological quality studies into five parts: qualitative research, randomised controlled trials, non-randomised studies, quantitative descriptive studies and mixed methods studies.^60^ MMAT was the only appraisal tool employed for mixed-method studies.^61^ MMAT assessment tool comprises two sections with embedded sub-questions. It includes two screening questions and different methodological quality criteria according to the category of study designs. All questions are to be answered with ‘yes’, ‘no’ or ‘can’t tell’. A ‘no’ or a ‘can't tell’ response to one or both screening questions could mean that the paper is not an empirical study and, hence, cannot be evaluated using the MMAT. It is unlikely to have numbered for ‘yes’ for each citation in the methodological quality criteria as the research team usually decides on what is important to include from their discipline.^60^

(4) Charting the data

Authors agreed with data to extract. The main reviewer, as part of their doctoral work, extracted papers. All authors reviewed and discussed a sample of the included and excluded papers. The approach of data charting was iterative. Authors employed Arkesy and O’Malley's ‘descriptive analysis’^53^ and Levac's recommendation for data extraction,^51^ summarising it in an Excel sheet, including the demographic data, intervention and tool and thematic approaches, such as the educational outcome and educational model.

(5) Collecting, summarising and reporting the results

As found in the literature, there was not a single model to synthesise IPE, SIM or VR. This scoping review adopted the aforementioned four models for the analysis and synthesis of VR-SIM IPE, as follows:

Interprofessional capabilities:

The Combined Universities Interprofessional Learning Unit (CUILU) framed the Interprofessional Capability Framework and identified sixteen capabilities in what is considered the oldest framework for interprofessional learning.^24,27^ CUILU is a joint effort between the University of Sheffield and Sheffield Hallam University.^27^

These capabilities aim to foster teamwork, partnership and collaboration between professionals and patients by providing a more coherent, integrate and patient-centred approach to upgrading educational input for health professionals.^24^ This framework has been thoroughly analysed and organised into four main domains categorised by the CUILU team, illustrated in Table 1.

Revised Bloom's Taxonomy:

Bloom's Taxonomy is a hierarchical concept that divides learning objectives into different difficulty levels. It consists of three types of learning: cognitive, emotional and psychomotor.^62^ The cognitive domain is the subject of this review. Benjamin Bloom published the first Bloom's taxonomy (original) in 1956. Forty-five years later, Anderson and Krathwohl produced an updated ‘Revised Taxonomy’ model.^63^

The Revised Bloom's Taxonomy knowledge and cognitive process dimension table comprises two dimensions: the knowledge dimension and the cognitive dimension. There are four levels of the knowledge dimension: factual, conceptual, procedural and metacognitive. There are six levels of the cognitive process dimension: remembering, understanding, applying, analysing, evaluating and creating,^34^ illustrated in Table 1. These levels allow educators to reflect more deeply on the material they are teaching and the focused goals. It also classifies goals more broadly and in a way that helps them understand the intricate connections between knowledge and cognitive processes.^34^

Learning efficacy:

To measure the learning efficacy of implementing the interprofessional reality simulation as a teaching strategy accurately, this review adopted the six-point learning efficacy scale to evaluate active learning.^36^ This learning strategy is based on the classification of the modiﬁed version of the GER strength of evidence pyramid,^36^ illustrated in Table 1.

The modified Kirkpatrick's model:

The Kirkpatrick's model is the most used evaluation model cited in academic research and is the most well-known applied paradigm for evaluations.^37^ The Kirkpatrick's model has encouraged the development of several new assessment models and transferred the focus of training assessment practice to the findings; thus, it is highly adapted in an education setting to measure training effectiveness accurately. This model can measure learning outcomes, such as knowledge acquisition, skill development, behavioural changes and patient outcomes, as well as other important programme-based outcomes, including OSCE performance^64^ and clinical entrustable professional activities (EPA) achievement.^65^

Therefore, in this review, the educational outcome will be categorised based on the four models,^38^ illustrated in Table 1.

**Figure 1.**
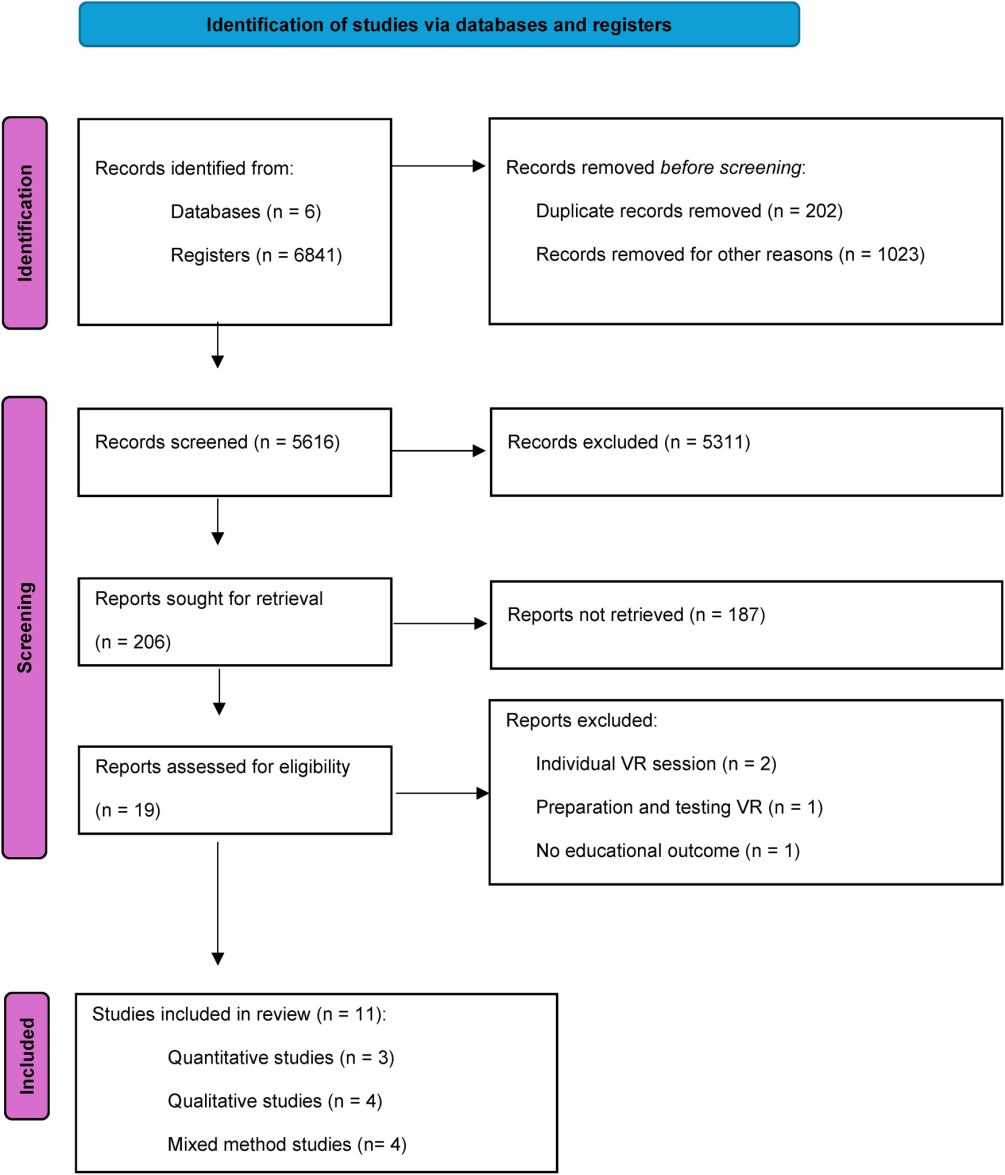
Scoping review 2010-2022 (PRISMA-ScR)^52^ chart.

**Figure 2.**
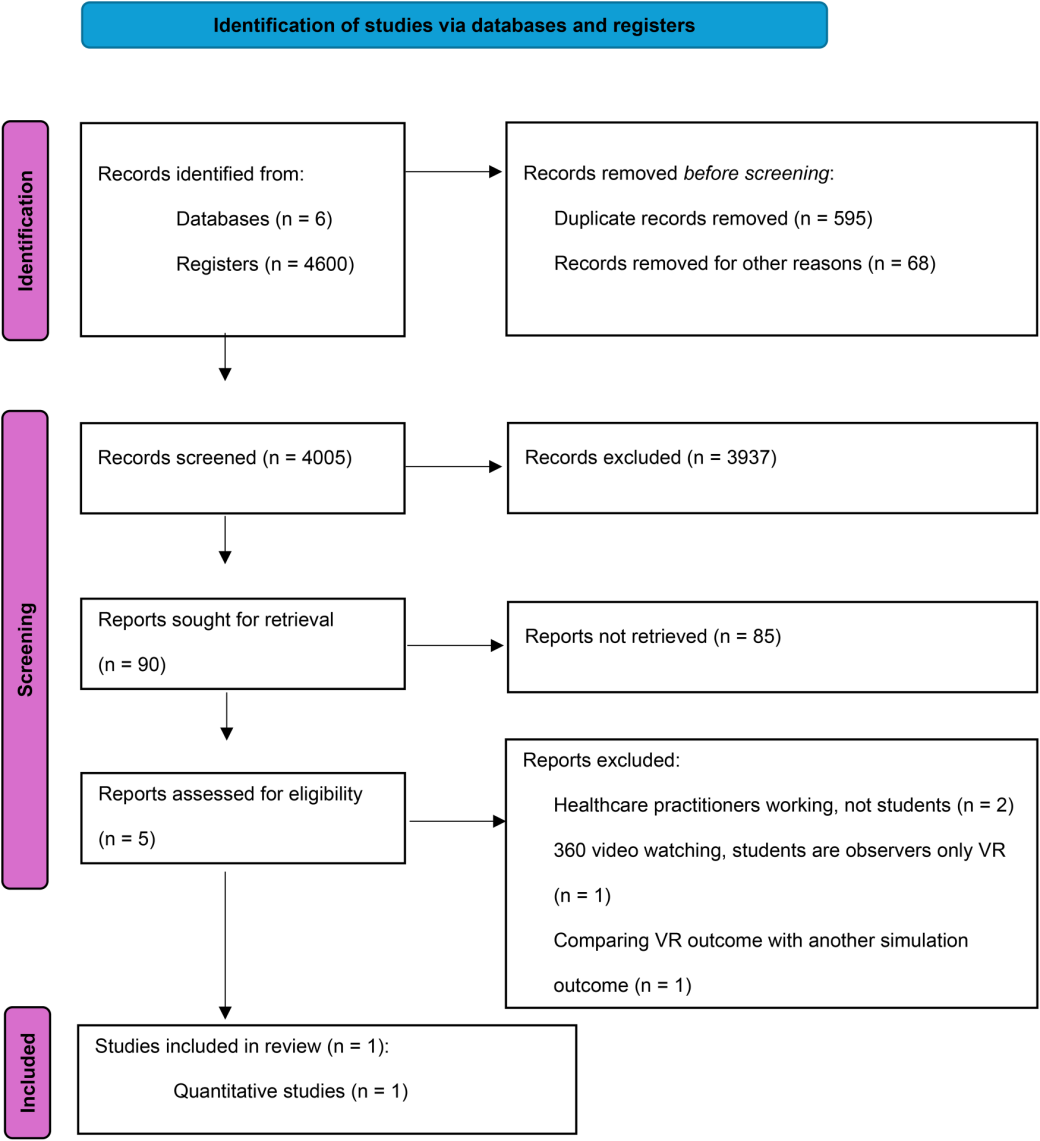
Scoping review 2022-February 2025 (PRISMA-ScR)^52^ chart.

Reporting results are presented in the (PRISMA-ScR) chart^52^ shown in Figure 1 for the search from 2010 to 2022, and Figure 2 for the search from 2022 to February 2025, and the detailed descriptive analysis is illustrated in Table 2.

## Results

### Selection of Sources of Evidence

#### Characteristics of included studies

The review covered 12 research paper from 2010 to 2025. Four of these were quantitative studies, all of them were quasi-excremental studies, ie, pre-post design was included,^66–69^ four qualitative studies^70–73^ and four mixed-method studies.^74–77^ Limited counties were included in these activities; half of the studies were in the USA (50%), followed by Singapore (16%) and a single study in each of the following countries: Canada, Sweden, New Zealand, and Switzerland.

Sampling differed in the size, professions and distribution of each discipline in research. The total sample sizes varied from 16 to 100 participants.^72,73^ On the other hand, the disciplines’ sample size ranged from 1 to 46 learners. The smallest sample size was for social worker learners,^73^ whereas the biggest sample size was for physiotherapy.^72^ Thirteen professions were involved in the review. The heterogeneous professions included were scientifically notable. Nursing was the most dominant profession, with around 83.3% of included articles, followed by medicine (75%). Social workers and physiotherapists were equally involved in 41.6% of the studies, followed by occupational therapy and pharmacy students at 25%. On the other hand, psychologists, speech therapists, health care assistants, physician assistants and nutritionist were the minor professional groups involved in the studies.

### VR-Sim IPE

A number of different virtual platforms were mentioned in the review, such as Sim X,^66^ the Second Life virtual simulation platform,^68,77^ the Clinisapce platform^70^ and the 3D-VE platform.^75^ The aforementioned platforms are a variety of three-dimensional multiplayer online/computer environments, sometimes referred to as ‘virtual worlds’. The number of VR sessions delivered also varied up with up to 20 scenarios/sessions.^74^ To measure the quantitative data, 16 validated instruments were used. Four of these instruments were utilised twice: Interprofessional Socialization and Valuing Scale (ISVS),^67,75^ TeamsSTEPPS Teamwork Attitudes Questionnaire (T-TAQ),^69,77^ Attitudes Towards Health Care Team Survey (ATHCT)^75,76^ and Readiness for Interprofessional Learning Scale (RIPLS).^74,76^ In contrast, nine other instruments were used only once in a single article, ie, Interdisciplinary Education Perception Scale (IEPS), Interprofessional Collaboration Scale (ICS), Team Skills Scale (TSS), Team Fitness Test (TFT), System Usability Scale (SUS), TeamworkSTEPPS Team Performance Observation Tool (T-POT), Technology and Overall Experience Survey (TOES), the sociability of computer-supported collaborative learning scale, the French-Canadian adaptation questionnaire, Handover Assessment Tool (ISBAR), self-constructed questionnaire and Training Evaluation Inventory**.** Only one study^68^ used their ten-item survey developed by IPE faculties. Table 2 shows a descriptive summary of each included study.

Characteristics of sources of evidence:

See Table 2.

## Discussion

From the literature, it was found that various health professionals used VR-Sim IPE in health teaching.^15^ The review showed a range of 13 professions, with the most common being nursing, followed by medicine. It would be beneficial to empower other allied health professionals to assess the effects of adopting VR-Sim IPE in their training, for example, psychology, speech therapy, health care assistants, physician assistants and nutrition. In terms of VR platforms, the review covered only four VR platforms: Sim X, Second Life, Clinispace and 3D-VE. The literature did not narratively discuss the types of VR platforms and their differences in features.

As the review scope is focused on assessing non-technical skills, 16 tools were found that assessed VR-Sim IPE outcomes. From these, four were utilised twice in the articles: ISVS, T-TAQ, ATHCT and RIPLS, and this promotes the facilitation and inclusion of the IPE VR in assessing the non-technical skills. From these tools, highly significant quantitative statistical P values were shown in the T-TAQ^69^ in leadership, situation monitoring, mutual support and communication, as well as with the RIPLS^74^ in teamwork and collaboration and positive professional identity. In terms of qualitative results, many positive themes emerged from the included articles.^70–77^ Learners better understood their own and other roles, boundaries and responsibilities, teamwork, collaboration and communication, equality of positions of power and developing a shared plan. Also, there was improved knowledge and competencies, understanding of patient needs and holistic care and decision-making. All these outcomes are covered in literature from the beginning of the definition and aim of interprofessional education^1^ through to considering how simulation enhances learning.^7–10,78,79^ This review directly links to VR specifically as a teaching tool in the field of IPE. However, VR showed drawbacks like any other technology may show drawbacks. The leading VR limitation in this review was delivering non-verbal communication, such as body language and facial expressions.^75,76^

The integration of the interprofessional capability framework, revised Bloom's Taxonomy**,** learning efficacy model and the modified Kirkpatrick's model offers a comprehensive approach to designing, implementing and evaluating VR-SIM IPE. Each model contributes unique strengths to fostering the development of collaborative skills and ensuring the effectiveness of IPE experiences within the VR-SIM context.

### VR-Sim IPE Capabilities

All review outcomes embraced the four IPE domains/capabilities, ie, knowledge in practice, ethical practice, interprofessional working and reflection (learning).^27^ Interestingly, two articles identified almost all IPE domains/capabilities.^68,71^ This is in line with the CUILU aims,^24,27^ and these capabilities were measured in a harm-free learning environment provided by the VR, similar to any other simulation typology that keeps patients safe and reduces the tension of cost and tension of clinical training.

### VR-Sim IPE Knowledge Cognitive Process Dimensions

This review used the knowledge cognitive process dimensions,^34^ to measure the impact of the chosen learning strategy and method on learners. Most of the included studies (42%) reported the analyse and conceptual knowledge dimensions level, and 33% reported the apply and conceptual knowledge level. Only three reports showed a higher cognitive process dimension: two studies (16.6%) captured the evaluate and conceptual knowledge level and another study (8.4%) captured the evaluate and procedural knowledge level, the highest level reported in the review. Absence of low level of cognitive dimensions, such as remembering and understanding level and the lowest level of knowledge dimensions, ie, factual knowledge, in addition to measuring such two high levels, ie, evaluate and conceptual knowledge level,^75^ and evaluate and procedural knowledge,^72^ indicate the strength of VR-Sim IPE in teaching. However, none of the articles mapped to create the cognitive level, as this could only be assessed in the actual hospital field. Metacognitive knowledge level was missed in the work; perhaps this requires more complex VR cases. However, more complex and diverse cases utilising the VR-Sim IPE are required and could be assessed in future studies.

### VR-Sim IPE Learning Efficacy

The 6-level learning efficacy model^36^ was used to map the studies. None of the studies were rated as low strengths of evidence (levels 1 and 2). Most of the studies (66.6%) were in level 4, ie, moderate strength of evidence, consistent of evidence learning gains in two or more settings or disciplines. A few were at the highest level, level 6, ie, high strength of evidence, consistent with evidence of learning gain in two or more settings or disciplines (33.3%).^66,72,75,76^ This indicates the positive impact of VR-Sim IPE activities in these institutions and is in line with previous suggestions and recommendations of utilising simulation in the healthcare field.^7–10^

### VR-Sim IPE Effectiveness of Learning Outcome

The modified Kirkpatrick's model for evaluating educational outcomes is one of the best evaluation models to evaluate educational outcomes.^38^ None of the articles was at the lowest level (reaction). It varied from level 2A, ie, change in attitudes (33%), to level 2B, ie, modification of knowledge or skills (25%) and level 3, ie, change in behaviours (42%),^66,68,72,75,76^ the highest level from the review. None of the research captured Level 4A and Level 4B, as these are more likely to be assessed in the hospital field. However, level 3, ie, changes in behaviour, is the highest outcome level that can be assessed in an academic setting, such as VR. Three out of five articles finding level 3^66,72,75^ are the same studies that captured higher knowledge cognitive dimensions levels as described previously in the review, ie, evaluate and conceptual knowledge level and evaluate and procedural knowledge. This shows the consistency of outcome measures that align different models at similar levels.

The review showed significant outcomes in measuring the effectiveness of VR-Sim IPE. These findings are aligned with transformative learning theory^43–45^ where learners showed non-technical skills acquisition. Three major studies^66,72,75^ reported positive outcomes across three models, the highest levels mentioned in the review for the learning efficacy, cognitive knowledge process dimension and the modified Kirkpatrick's level.

### VR-Sim IPE Practical Education Application

From this scoping review findings, VR has successfully provided a practical education application in the field of IPE, particularly in non-technical skills. Studies have demonstrated that VR-Sim IPE can enhance the development of collaborative skills.

For example, a study^75^ utilised a deteriorating VR patient to enhance teamwork and decision-making among nursing and medical students, demonstrating advancements in both role clarity and team-based responses. Similarly, in another study^67^ simulated a VR cardiac arrest case scenario, assisting learners in developing collaborative leadership skills and effectively delegating roles in high-pressure situations. From ten complex VR clinical tasks, a study^71^ demonstrated an understanding of the roles and competencies of various professions, including nursing, medicine, physiotherapy and occupational therapy. Moreover, another study^68^ facilitated mutual understanding and communication during patient care handoffs between nursing and respiratory therapy students.

Research underscores the capacity of VR to enhance both empathy and teamwork by immersing learners in realistic, patient-centred scenarios that necessitate collaborative interprofessional efforts. Scholars^72^ have developed a VR environment that allows learners to explore patient experiences, fostering empathy and shared values among team members. Another scholar^77^ also showed that a VR environment could enhance attitudes toward team-based care, particularly in complex geriatric scenarios.

Additional studies have shown that VR enhances clinical judgment and teamwork during interprofessional ward rounds and transitional care planning. A VR ward-round model^70^ was implemented to enhance interprofessional teamwork in discharge planning, resulting in improved communication efficiency and a stronger patient-centred focus. Another research study^74^ shared positive results on medication reconciliation and discharge planning, highlighting how VR enhanced interprofessional collaboration.

Moreover, another study^69^ examined the use of VR in structured interprofessional curricula. The study found that VR can be effectively integrated into team-based simulation programmes, fostering shared mental models and enhancing real-time collaborative problem-solving during clinical deterioration or transitions of care.

These applications illustrate how VR not only creates engaging learning experiences but also bridges theory and practice in IPE by replicating the complexities of real-world clinical environments. Importantly, VR supports experiential learning in safe, repeatable settings, which is particularly useful for novice learners. A study^73^ highlighted the use of VR to prepare students for interprofessional clinical placements, reducing anxiety and increasing preparedness for collaborative tasks.

Other researchers^76^ explored the application of VR in emergency obstetric scenarios, showing that students across different fields can participate in high-stress training without the dangers present in real clinical settings. Together, these studies suggest that VR improves both technical and non-technical skills while fostering critical reflection, empathy and teamwork in various healthcare environments.

Finally, the most recent article^66^ included in the review indicates that managing VR seizure patients within an IPE team enhances learners’ communication and handover abilities while also deepening their understanding of the roles and responsibilities of their peers from different disciplines. Students reported feeling increased confidence and capability following the training, highlighting how VR can effectively bridge the gap between theoretical education and practical clinical judgment.

### Limitations and Strengths of This Scoping Review

IPE VR is a wide topic for review. Considering the time and manpower, a concise methodology was needed to resolve the tension between conducting a comprehension systematic review and time restriction. For example, inclusion criteria were limited to health professions and non-technical skills. Only studies published in English were included, which may have excluded potentially relevant studies published in other languages. Grey Literature, conference papers and unpublished literature were excluded, which may have contained relevant unpublished studies, reports or theses. We searched the largest and most commonly used databases in health education research, ie, Web of Science, ProQuest, OVID, SCOPUS, CINAHL Plus, and British Educational Index BEI, for the last 15 years, which gives strength to the review.

### Recommendations for Future Work

Interprofessional education using VR should be implemented in undergraduate health students’ modules and used to assess the technical skills and algorithm of a procedure. VR can be utilised in assessment, for example, formative or summative OSCEs. Using longitudinal studies to assess the long-term impact of VR simulation on interprofessional collaboration skills must be considered. These would be very beneficial for health education outcomes and better patient care. Also, a broader range of health professions and non-health related professions should be included in the studies.

## Conclusion

The review followed the PRISMA-ScR protocol and demonstrated the usability of VR-Sim IPE; the participants varied in sampling size and heterogeny of profession included. It shows the VR-Sim IPE as an effective teaching modality since the review examined the results using four well-known models for syntheses: Interprofessional capabilities, cognitive process dimension from the modified Bloom's taxonomy, learning efficacy and the modified Kirkpatrick's model for evaluating educational outcomes. Also, each retrieved article was scanned for critical appraisal using high-quality assessment tools; MERSQI was used for quantitative studies, CASP was used for qualitative studies and MMAT was used for Mixed Methods studies. None of the articles assessed a procedure's technical skills or algorithm. In conclusion, virtual reality simulation for interprofessional education shows high effectiveness in improving non-technical skills among various educational models.

## Supplemental Material

sj-docx-1-mde-10.1177_23821205251346327 - Supplemental material for Scoping Review: The Effectiveness of Interprofessional Virtual Reality Simulation Supplemental material, sj-docx-1-mde-10.1177_23821205251346327 for Scoping Review: The Effectiveness of Interprofessional Virtual Reality Simulation by Nebras Alghanaim, Jo Hart and Gabrielle Finn in Journal of Medical Education and Curricular Development

sj-docx-2-mde-10.1177_23821205251346327 - Supplemental material for Scoping Review: The Effectiveness of Interprofessional Virtual Reality Simulation Supplemental material, sj-docx-2-mde-10.1177_23821205251346327 for Scoping Review: The Effectiveness of Interprofessional Virtual Reality Simulation by Nebras Alghanaim, Jo Hart and Gabrielle Finn in Journal of Medical Education and Curricular Development
