# Supplementary material for: Scoping Review: The Effectiveness of Interprofessional Virtual Reality Simulation
Source: J Med Educ Curric Dev. 2025 Jul 17;12:23821205251346327. doi: 10.1177/23821205251346327 (PMC12276473; doi:10.1177/23821205251346327)
Supplement: sj-docx-2-mde-10.1177_23821205251346327 - Supplemental material for Scoping Review: The Effectiveness of Interprofessional Virtual Reality Simulation [file sj-docx-2-mde-10.1177_23821205251346327.docx]

Your Journals@Ovid

Books@Ovid <February 14, 2022>

EBM Reviews - Cochrane Database of Systematic Reviews <2005 to February 16, 2022>

EBM Reviews - ACP Journal Club <1991 to January 2022>

EBM Reviews - Database of Abstracts of Reviews of Effects <1st Quarter 2016>

EBM Reviews - Cochrane Clinical Answers <January 2022>

EBM Reviews - Cochrane Central Register of Controlled Trials <January 2022>

EBM Reviews - Cochrane Methodology Register <3rd Quarter 2012>

EBM Reviews - Health Technology Assessment <4th Quarter 2016>

EBM Reviews - NHS Economic Evaluation Database <1st Quarter 2016>

AMED (Allied and Complementary Medicine) <1985 to February 2022>

Econlit <1886 to February 10, 2022>

Global Health Archive <1910 to 1972>

Health and Psychosocial Instruments <1985 to January 2022>

International Pharmaceutical Abstracts <1970 to February 2022>

Maternity & Infant Care Database (MIDIRS) <1971 to February 1, 2022>

APA PsycBooks <1806 to January 2022>

APA PsycInfo <1806 to February Week 2 2022>

Social Policy and Practice <202201>

APA PsycArticles Full Text

Embase <1974 to 2022 February 17>

HMIC Health Management Information Consortium <1979 to November 2021>

Ovid MEDLINE(R) and Epub Ahead of Print, In-Process, In-Data-Review & Other Non-Indexed Citations, Daily and Versions(R) <1946 to February 17, 2022>

Zoological Record <1978 to 2013>

1 Undergrad*.mp. [mp=ti, ot, ab, tx, ct, sh, bt, kw, de, md, sd, hw, id, cc, ac, ip, vo, pg, jn, yr, dp, pu, ib, is, et, ey, cd, cl, pb, sa, ja, bd, st, dg, dt, rf, so, pa, pi, pl, ry, mo, op, os, ar, bs, cf, pj, rw, nm, tn, tc, tm, mf, pt, an, ui, dm, dv, kf, fx, dq, ox, px, rx, ds, on, sy] 352026

2 Postgrad*.mp. [mp=ti, ot, ab, tx, ct, sh, bt, kw, de, md, sd, hw, id, cc, ac, ip, vo, pg, jn, yr, dp, pu, ib, is, et, ey, cd, cl, pb, sa, ja, bd, st, dg, dt, rf, so, pa, pi, pl, ry, mo, op, os, ar, bs, cf, pj, rw, nm, tn, tc, tm, mf, pt, an, ui, dm, dv, kf, fx, dq, ox, px, rx, ds, on, sy] 96571

3 1 or 2 434012

4 Interprofession*.mp. [mp=ti, ot, ab, tx, ct, sh, bt, kw, de, md, sd, hw, id, cc, ac, ip, vo, pg, jn, yr, dp, pu, ib, is, et, ey, cd, cl, pb, sa, ja, bd, st, dg, dt, rf, so, pa, pi, pl, ry, mo, op, os, ar, bs, cf, pj, rw, nm, tn, tc, tm, mf, pt, an, ui, dm, dv, kf, fx, dq, ox, px, rx, ds, on, sy] 103883

5 interprofessional learning.mp. [mp=ti, ot, ab, tx, ct, sh, bt, kw, de, md, sd, hw, id, cc, ac, ip, vo, pg, jn, yr, dp, pu, ib, is, et, ey, cd, cl, pb, sa, ja, bd, st, dg, dt, rf, so, pa, pi, pl, ry, mo, op, os, ar, bs, cf, pj, rw, nm, tn, tc, tm, mf, pt, an, ui, dm, dv, kf, fx, dq, ox, px, rx, ds, on, sy] 3514

6 interprofessional collaboration.mp. [mp=ti, ot, ab, tx, ct, sh, bt, kw, de, md, sd, hw, id, cc, ac, ip, vo, pg, jn, yr, dp, pu, ib, is, et, ey, cd, cl, pb, sa, ja, bd, st, dg, dt, rf, so, pa, pi, pl, ry, mo, op, os, ar, bs, cf, pj, rw, nm, tn, tc, tm, mf, pt, an, ui, dm, dv, kf, fx, dq, ox, px, rx, ds, on, sy] 9518

7 Health profession*.mp. [mp=ti, ot, ab, tx, ct, sh, bt, kw, de, md, sd, hw, id, cc, ac, ip, vo, pg, jn, yr, dp, pu, ib, is, et, ey, cd, cl, pb, sa, ja, bd, st, dg, dt, rf, so, pa, pi, pl, ry, mo, op, os, ar, bs, cf, pj, rw, nm, tn, tc, tm, mf, pt, an, ui, dm, dv, kf, fx, dq, ox, px, rx, ds, on, sy] 291141

8 4 or 5 or 6 or 7 384291

9 Simulation based.mp. [mp=ti, ot, ab, tx, ct, sh, bt, kw, de, md, sd, hw, id, cc, ac, ip, vo, pg, jn, yr, dp, pu, ib, is, et, ey, cd, cl, pb, sa, ja, bd, st, dg, dt, rf, so, pa, pi, pl, ry, mo, op, os, ar, bs, cf, pj, rw, nm, tn, tc, tm, mf, pt, an, ui, dm, dv, kf, fx, dq, ox, px, rx, ds, on, sy] 25228

10 interprofessional simulation.mp. [mp=ti, ot, ab, tx, ct, sh, bt, kw, de, md, sd, hw, id, cc, ac, ip, vo, pg, jn, yr, dp, pu, ib, is, et, ey, cd, cl, pb, sa, ja, bd, st, dg, dt, rf, so, pa, pi, pl, ry, mo, op, os, ar, bs, cf, pj, rw, nm, tn, tc, tm, mf, pt, an, ui, dm, dv, kf, fx, dq, ox, px, rx, ds, on, sy] 768

11 interprofessional virtual reality simulation.mp. [mp=ti, ot, ab, tx, ct, sh, bt, kw, de, md, sd, hw, id, cc, ac, ip, vo, pg, jn, yr, dp, pu, ib, is, et, ey, cd, cl, pb, sa, ja, bd, st, dg, dt, rf, so, pa, pi, pl, ry, mo, op, os, ar, bs, cf, pj, rw, nm, tn, tc, tm, mf, pt, an, ui, dm, dv, kf, fx, dq, ox, px, rx, ds, on, sy] 4

12 9 or 10 or 11 25685

13 Virtual reality.mp. [mp=ti, ot, ab, tx, ct, sh, bt, kw, de, md, sd, hw, id, cc, ac, ip, vo, pg, jn, yr, dp, pu, ib, is, et, ey, cd, cl, pb, sa, ja, bd, st, dg, dt, rf, so, pa, pi, pl, ry, mo, op, os, ar, bs, cf, pj, rw, nm, tn, tc, tm, mf, pt, an, ui, dm, dv, kf, fx, dq, ox, px, rx, ds, on, sy] 64117

14 Virtual simulation.mp. [mp=ti, ot, ab, tx, ct, sh, bt, kw, de, md, sd, hw, id, cc, ac, ip, vo, pg, jn, yr, dp, pu, ib, is, et, ey, cd, cl, pb, sa, ja, bd, st, dg, dt, rf, so, pa, pi, pl, ry, mo, op, os, ar, bs, cf, pj, rw, nm, tn, tc, tm, mf, pt, an, ui, dm, dv, kf, fx, dq, ox, px, rx, ds, on, sy] 2195

15 13 or 14 65756

16 Team based competenc*.mp. [mp=ti, ot, ab, tx, ct, sh, bt, kw, de, md, sd, hw, id, cc, ac, ip, vo, pg, jn, yr, dp, pu, ib, is, et, ey, cd, cl, pb, sa, ja, bd, st, dg, dt, rf, so, pa, pi, pl, ry, mo, op, os, ar, bs, cf, pj, rw, nm, tn, tc, tm, mf, pt, an, ui, dm, dv, kf, fx, dq, ox, px, rx, ds, on, sy] 89

17 Interprofessional competenc*.mp. [mp=ti, ot, ab, tx, ct, sh, bt, kw, de, md, sd, hw, id, cc, ac, ip, vo, pg, jn, yr, dp, pu, ib, is, et, ey, cd, cl, pb, sa, ja, bd, st, dg, dt, rf, so, pa, pi, pl, ry, mo, op, os, ar, bs, cf, pj, rw, nm, tn, tc, tm, mf, pt, an, ui, dm, dv, kf, fx, dq, ox, px, rx, ds, on, sy] 850

18 Skill*.mp. [mp=ti, ot, ab, tx, ct, sh, bt, kw, de, md, sd, hw, id, cc, ac, ip, vo, pg, jn, yr, dp, pu, ib, is, et, ey, cd, cl, pb, sa, ja, bd, st, dg, dt, rf, so, pa, pi, pl, ry, mo, op, os, ar, bs, cf, pj, rw, nm, tn, tc, tm, mf, pt, an, ui, dm, dv, kf, fx, dq, ox, px, rx, ds, on, sy] 1295078

19 16 or 17 or 18 1295605

20 3 and 8 and 12 and 15 and 19 75

21 limit 20 to english language 75

22 limit 21 to yr="2010 -Current" 68
